# Physiological Age and Homeostatic Dysregulation Following Child Maltreatment in Youth

**DOI:** 10.1038/s41380-026-03642-z

**Published:** 2026-05-08

**Authors:** Qiaofeng Ye, Abner T. Apsley, Laura Etzel, Waylon J. Hastings, Christopher R. Chiaro, Hannah M. C. Schreier, Eric D. Claus, Alan A. Cohen, Zachary Fisher, Christine M. Heim, Jennie G. Noll, Chad E. Shenk, Idan Shalev

**Affiliations:** 1 Department of Biobehavioral Health, The Pennsylvania State University, University Park, PA 16802 USA; 2 National Science Foundation Science and Technology Center for Quantitative Cell Biology, Beckman Institute for Advanced Science and Technology, University of Illinois at Urbana–Champaign, Urbana, IL 61801 USA; 3 Department of Nutrition, Texas A&M University, College Station, TX 77843 USA; 4 Institute for Advancing Health Through Agriculture, Texas A&M AgriLife, College Station, TX 77843 USA; 5 Department of Environmental Health Sciences and Robert N. Butler Columbia Aging Center, Columbia University Mailman School of Public Health, New York, NY 10032 USA; 6 Department of Psychology and Neuroscience, The University of North Carolina at Chapel Hill, Chapel Hill, NC 27599 USA; 7 Charité – Universitätsmedizin Berlin, corporate member of Freie Universität Berlin and Humboldt-Universität zu Berlin, Institute of Medical Psychology, Berlin, 10117 Germany; 8 Charité – Universitätsmedizin Berlin, corporate member of Freie Universität Berlin and Humboldt-Universität zu Berlin, NeuroCure Cluster of Excellence, Berlin, 10117 Germany; 9 German Center for Mental Health, Berlin Potsdam partner site, Berlin, 10117 Germany; 10 Department of Psychology, University of Rochester, Rochester, NY 14627 USA; 11 Mt. Hope Family Center, University of Rochester, Rochester, NY 14627 USA

**Keywords:** Physiology, Predictive markers

## Abstract

Child maltreatment has been associated with biological hallmarks of aging, including telomere shortening and epigenetic instability; however, its influence on physiological age and homeostatic dysregulation in early life remains unclear. The current study examined pediatric versions of physiological age and homeostatic dysregulation in children aged 8-13 with and without exposure to maltreatment. Maltreatment exposure was determined based on investigational records within 12 months prior to enrollment. Physiological measures were trained and validated utilizing external datasets — the National Health and Nutrition Examination Survey III and IV, respectively. Physiological age was computed using the Klemera-Doubal Method to indicate physiological developmental status. Homeostatic dysregulation level was computed as the Mahalanobis distance from an external reference group. 216 females and 245 males with a mean age of 11.4 years (SD 1.5) were included (76.6% White, 13.2% Black, and 13.0% Hispanic, 76.6% with maltreatment). Exposure to maltreatment was not associated with changes in physiological age but was associated with greater homeostatic dysregulation. Further analyses by maltreatment type and sex revealed that physical abuse was associated with greater homeostatic dysregulation, while sexual abuse was associated with delayed physiological development, specifically in males. Exposure to multiple types of maltreatment was associated with greater homeostatic dysregulation among males, but not among females. This study revealed that recent exposure to certain types of maltreatment may impair physiological development or regulation in children, with sex-specific patterns suggesting greater effects in males. Findings further indicate that physiological development composites in youth are sensitive to the impact of child maltreatment and can be incorporated in future work to evaluate the long-term sequelae of adverse exposures in pediatric populations. As the impact of maltreatment was only nominally significant after correction for multiple testing, validation work in other samples is needed.

## Introduction

Child maltreatment is a potent early-life stressor that can lead to negative health consequences lasting into adulthood. Negative outcomes of child maltreatment may include dysregulated neuro-endocrine stress response [1], compromised general physical health [2], increased risky health behaviors [1], elevated risk of obesity [3], disproportionate mental health burden [2, 4], and premature mortality [5]. Child maltreatment has a high prevalence in the United States; 37.4% of youth under 18 years are investigated for potential exposure to maltreatment [6], with 12.5% being substantiated cases [7]. While child maltreatment is of great public health concern, the biological mechanisms linking child maltreatment to negative health outcomes are not yet fully understood [8]. Biological pathways linking child maltreatment to adverse health outcomes have attracted substantial research interest in recent years, with systemic inflammation [9], epigenetic reprogramming [10], and accelerated development and cellular aging [11] emerging as plausible mechanisms.

Biological development and aging form an interconnected continuum throughout an individual’s life, the trajectory of which can be shaped by early life experiences [12]. The emergence of various biological age quantification methods facilitates research on this continuum [13]. Physiological composites of anthropometric and blood-chemistry biomarkers have emerged as tools to measure biological developmental and aging trajectories among individuals, and can be studied beginning in early life [12]. Klemera-Doubal Method (KDM) is a physiological composite originally developed to measure biological aging in adult populations and has been shown to predict mortality more accurately than chronological age [14]. KDM assumes physiological changes of aging develop continually and is calculated using parameters estimated from a series of regressions of individual biomarkers on chronological age in a reference population. KDM prediction corresponds to the chronological age at which an individual’s physiology would be normal in the reference population [14, 15]. An alternative physiological composite, known as homeostatic dysregulation (HD), was developed based on the idea that aging involves the gradual breakdown of complex physiological systems responsible for maintaining homeostasis [16, 17]. HD quantifies systemic dysregulation using Mahalanobis distance, the multivariate distance of multiple biomarkers from their joint distribution in a reference population. HD has been shown to increase with chronological age and can predict higher risk of mortality in adults [16, 18]. However, neither KDM nor HD has been applied in pediatric populations. Direct application of KDM or HD in pediatric populations is not appropriate because the relationships of physiological biomarkers with chronological age differ between children and adults. Therefore, it is necessary to adapt the two methods by using a pediatric reference population for parameter estimation as well as selecting a new set of biomarkers appropriate for pediatric usage.

Previous studies have shown that early life adversity is associated with accelerated cellular aging as measured by DNA methylation clocks and telomere length [11], but few studies have investigated the impact of child maltreatment on physiological age and HD. Specifically, prior studies reported that exposure to maltreatment in childhood was significantly associated with older KDM physiological age and greater HD in adulthood [19, 20], but it is unknown whether this association begins in childhood or manifests only in adulthood. Comparisons among various approaches to quantifying biological aging suggest they may be measuring different aspects of development and the aging process [21, 22]. Thus, evidence of the impact of child maltreatment on physiological development and HD would be informative for developing interventions to minimize the negative long-term health consequences of maltreatment exposure.

The current study aimed to examine the impact of maltreatment exposure on pediatric versions of physiological age and HD among children aged 8-13 years, as measured by KDM and Mahalanobis distance, respectively.

## Materials and Methods

### Study design, participant recruitment, and assessment procedure

Child participants were members of the Child Health Study (CHS, P50HD089922), a large multidisciplinary study designed to provide prospective, longitudinal data on the health and development of children with and without a history of maltreatment investigations (for more details about the Child Health Study see [23]). Approval from The Pennsylvania State University Institutional Review Board was granted (protocol STUDY00006550), and informed assent (child) and consent (caregiver) was obtained from all participants. All methods were performed in accordance with the relevant guidelines and regulations. Children with a history of past-year maltreatment investigation (i.e., defined under Pennsylvania state law to include physical abuse, sexual abuse, and neglect) were identified and recruited using county Child Welfare Information Systems data from participating Pennsylvania counties. Demographically matched comparison children were recruited across Pennsylvania through advertisements on local print media, social media, flyers at community centers, pools, electronic school list serves, and local radio stations. Families who expressed interest contacted the research center and consented to an administrative record review to confirm no lifetime history of child welfare involvement. Children were aged 8-13 years at recruitment and were invited to visit the assessment center every two years. Recruitment and Visit 1 assessment were completed for all the participants (*N* = 700) by the year 2024. Visit 1 data were used for the current analyses.

Families arrived at the research facility at 7:30 am for their visit, after having stayed the previous night at a local hotel. Following an introduction to the study and providing informed assent and consent, fasting whole blood was collected from the child via antecubital venipuncture by a trained phlebotomist followed by a physical exam. Blood biomarkers were measured by Quest Diagnostics within 24 hours of collection using blood collected in serum separator and EDTA tubes. Following the child’s blood draw, participating families received breakfast on-site and participated in the remaining study procedures including multiple questionnaires for the caregiver and child.

Study data were collected and managed using REDCap electronic data capture tools hosted at Penn State Health Milton S. Hershey Medical Center and Penn State College of Medicine. REDCap (Research Electronic Data Capture) [24] is a secure, web-based application designed to support data capture for research studies, providing an intuitive interface for validated data entry, audit trails for tracking data manipulation and export procedures, automated export procedures for seamless data downloads to common statistical packages, and procedures for importing data from external sources.

### Child maltreatment measures

Child maltreatment exposure was determined based on county Child Welfare Information Systems records of investigation. Types of child maltreatment included physical abuse, sexual abuse, and neglect. Polyvictimization was defined as a child being investigated for multiple maltreatment types. These maltreatment exposure variables were used for our main analyses.

In addition, exploratory analyses used more detailed information of maltreatment exposure. For a subset of the participants for whom consent for access to their county welfare data was available for purposes other than recruitment, a comprehensive classification index of lifetime maltreatment exposure before Visit 1 was created using the Cicchetti-Manly Maltreatment Classification System (MCS) [25]. Each individual record was independently evaluated by two trained coders (including the study PI; HS) and any discrepancies in ratings were resolved via team discussion and joint record review. Coders erred on the side of coding records conservatively, i.e., not relying solely on ‘hearsay’ information in the record but taking into account whether the record offered any supporting information or corroborating reports from other individuals. Thus, each record was coded as providing or not providing evidence of likely child maltreatment (“0” = “no child maltreatment,” “1” = “child maltreatment”); additionally, for any record coded as “1” in the first step, the presence (“1”) or absence (“0”) of each of the following subtypes was determined: physical abuse, sexual abuse, physical neglect – failure to provide, physical neglect – lack of supervision, emotional maltreatment, moral/legal maltreatment, and educational maltreatment. Number of coded incidences of each maltreatment type was counted to indicate frequency. The temporal distance between the Visit 1 date and the first report date of each maltreatment type was calculated to indicate the years since the first report. Both the frequency and years since the first report were used in exploratory analyses. Based on the distribution of the two variables, frequency was categorized to 0, 1, and 2 or more, and years since the first report was categorized to no exposure, >0 to <2 years, and 2 years or longer.

### Physiological age and homeostatic dysregulation

#### Reference population

The calculation of KDM physiological age and HD entails an external reference population. Participants aged 8-14 years involved in the National Health and Nutrition Examination Survey (NHANES) III (1988-1994) [26] were selected to form a reference population (Supplementary Table 1). NHANES III participants were matched with CHS participants by age, sex, race, and ethnicity (Supplementary Fig. 1). Matching weights were derived and used for analyses.

#### Biomarker selection

Overlaps of physiological biomarkers were checked between the CHS and NHANES III cohorts. In total, twenty-seven blood-based and anthropometric biomarkers were measured in both CHS and NHANES III. These biomarkers were subject to a three-step down-selection process. First, biomarker distributions were checked for consistency between NHANES III and CHS (Supplementary Fig. 2). Biomarkers with inconsistent distributions between the two cohorts were excluded. Second, biomarkers with a Pearson’s correlation with chronological age below 0.1 were excluded [22] (Supplementary Fig. 3). Third, correlations between biomarkers were computed (Supplementary Figs. 4&5), and consistent with prior research [22], one biomarker from each pair with a Pearson’s correlation greater than 0.4 was excluded. In total, 9 biomarkers were kept in the panel, including lymphocyte percentage, mean corpuscular volume, red cell distribution width, hemoglobin, platelet count, total cholesterol, high-density lipoprotein cholesterol, systolic blood pressure, and heart rate. Blood biomarkers in the NHANES III were measured in laboratories with strict routine quality control including inserting quality control specimens in each analytical run to monitor daily analyses [27]. Blood biomarkers in the CHS were measured by Quest Diagnostics, which is a national platform offering laboratory tests to doctors, patients, and researchers and has accreditation from regulatory agencies and accrediting organizations such as Center for Medicare and Medicaid Services, and College of American Pathologists [28].

#### Klemera-Doubal Method of physiological age measure

KDM was calculated from an algorithm with parameters estimated from a series of regressions of individual biomarkers on chronological age in the reference group [14, 29]. KDM prediction corresponds to the chronological age at which an individual’s physiology would be normal in the reference. The formula used to calculate KDM was: ${{BA}}_{E}=\frac{{\sum }_{j=1}^{m}\left({x}_{j}-{q}_{j}\right)\frac{{k}_{j}}{{s}_{j}^{2}}}{{\sum }_{j=1}^{m}{\left(\frac{{k}_{j}}{{s}_{j}}\right)}^{2}}$, where m is the number of biomarkers, q is the regression intercept of each biomarker regressed on chronological age, k is regression slope of each biomarker regressed on chronological age, and s is the root mean squared error of each regression. Regressions to estimate KDM parameters were performed independently in males and females.

#### Physiological Homeostatic Dysregulation

HD was calculated for the values of a set of biomarkers relative to the mean values of the biomarkers in the reference group [17, 29]. HD prediction in this study indicates the difference of an individual’s physiology from the reference. The formula is: ${HD}=\sqrt{{\left(\vec{x}-\vec{\mu }\right)}^{T}{S}^{-1}\left(\vec{x}-\vec{\mu }\right)}$, where $\vec{x}$ is a vector with all the biomarker values for an individual, $\vec{\mu }$ is a vector of mean values of each biomarker in the reference, and *S* is a variance-covariance matrix of biomarkers in the reference. The HD value was then natural log-transformed and scaled to have a standard deviation of 1. HD was estimated independently in males and females.

### Other measures

Demographic information—including chronological age, sex, race, ethnicity, household income, and number of people supported by the household income—was collected via caregiver reports and controlled in analyses to account for potential confounding in the relationship between maltreatment and physiological age or HD. Sex was coded as male or female. Two participants who identified as transgender were excluded. Race was coded as White, Black or African American, and other. Ethnicity was coded as non-Hispanic or Hispanic. Household income was reported by caregivers as current total household family income before taxes and was categorized into brackets of $0-$39,999, $40,000-$79,999, and above $80,000. Number of people supported by the household income was categorized into 1-3 people, 4-6 people, and 7 or more people. Body mass index was defined as body mass divided by squared body height. Body mass and body height of the participants were measured by trained CHS staff. Age- and sex-specific BMI percentiles were further derived using the Center for Disease Control’s BMI Calculator.

Pubertal development was assessed via self-report using the picture-assisted Tanner Staging Scale of secondary sex characteristics. Under sensitive guidance, participants reported the stage that most closely resembled their own body. Male participants reported on stage of genitalia and pubic hair development; female participants reported on stage of breast and pubic hair development. The developmental stage of each sex characteristic was determined on a scale of 1 to 5: stage 1 (pre-pubertal), stages 2-4 (pubertal), and stage 5 (post-pubertal). Ratings based on pubic hair and genitalia or breast were then averaged. The averaged Tanner stage rating was used in the analyses.

### Statistical analysis

All statistical analyses were performed using R 4.3.2. Biomarker outliers (<Q1-1.5*IQR or >Q3 + 1.5*IQR; Q1&Q3 are lower and upper quartiles; IQR is interquartile range) were winsorized to the boundary value of the range (i.e., Q1-1.5*IQR or Q3 + 1.5*IQR) (Supplementary Figs. 6&7). The percentage of data winsorized of included biomarkers ranged from 0.5% to 2.7% in NHANES III and 0.6% to 2.3% in CHS. KDM and HD were calculated using functions modified from the *BioAge* package [29]. KDM and HD values were next residualized for chronological age and z-standardized separately in males and females, and used as the outcome measures for analyses. Participants who did not consent for blood draw, with any missing data on demographic characteristics or biomarkers, or who were not matched with individuals in the reference group were excluded from analyses (*N* = 237). Comparison of participants excluded and included can be found in Supplementary Table 2. Excluded participants were younger, more likely female, or of non-White race. Sociodemographic variables, including chronological age, sex, race, ethnicity, family income, number of people in the household, and BMI percentile, were compared between the maltreatment and comparison groups. Continuous variables including chronological age and BMI percentile were tested for normality in each group using the Shapiro-Wilk test. For variables significantly deviating from normality, the Wilcoxon rank sum test was used to test the difference between groups. Otherwise, a two-sample t-test was used. The Bartlett test was used to test the homogeneity of variances across groups to choose a t-test with equal or unequal variances. For categorical variables including sex, race, ethnicity, family income, and number of people in the household, the Pearson’s Chi-squared test was used if expected cell counts were all greater than 5, or else, the Fisher’s exact test was used to test the difference between groups. As the CHS cohort included siblings (52 families with siblings), generalized estimating equation (GEE) models were employed to control for the nested data structure. The *geepack* package [30] was used to estimate GEE model parameters. Exchangeable correlation structure was assumed for GEE models, and robust estimators of standard error were used. Analyses involving maltreatment exposure were controlled for sex, race, ethnicity, household income, number of people in the household supported by the income, and BMI percentile. Sex differences were evaluated using interaction terms within a single model per outcome to formally test effect modification while limiting the number of inferential tests. To address multiplicity, false discovery rate control (Benjamini–Hochberg method) was applied within each outcome across maltreatment main effects and interaction terms (9 inferential tests per outcome). Findings are interpreted in light of both raw and adjusted p-values.

## Results

### CHS Cohort Characteristics

Analyses included 461 children with a mean chronological age of 11.4 years (SD 1.5), among which 216 (46.85%) were females, 353 (76.57%) identified as White, 61 (13.23%) Black, 60 (13.02%) Hispanic, and 353 (76.57%) had experienced maltreatment. The group with a history of child maltreatment investigation had older chronological age, higher percentage of Hispanic ethnicity, lower family income, and higher BMI percentile than the comparison group (Table 1).

**Table 1:** Demographic characteristics of the CHS cohort (*N* = 461).

Variable	Statistical Type/Level	All (*N* = 461)	Maltreatment status	
			Comparison (*N* = 108)	Maltreatment (*N* = 353)
Age	Mean (SD)	11.4 (1.5)	11.0 (1.5)	11.6 (1.5)^a^
	Min / Max	8.13 / 13.99	8.13 / 13.91	8.27 / 13.99
Sex	Female	216 (46.85%)	49 (45.37%)	167 (47.31%)
	Male	245 (53.15%)	59 (54.63%)	186 (52.69%)
Race	White	353 (76.57%)	86 (79.63%)	267 (75.64%)
	Black or African American	61 (13.23%)	12 (11.11%)	49 (13.88%)
	Other	47 (10.20%)	10 (9.26%)	37 (10.48%)
Ethnicity	Non-Hispanic	401 (86.98%)	101 (93.52%)	300 (84.99%)^a^
	Hispanic	60 (13.02%)	7 (6.48%)	53 (15.01%)^a^
Income	$0-$39,999	234 (50.76%)	30 (27.78%)	204 (57.79%)^a^
	$40,000-$79,999	130 (28.20%)	36 (33.33%)	94 (26.63%)^a^
	$80,000 or above	97 (21.04%)	42 (38.89%)	55 (15.58%)^a^
N of people in the household	1-3 people	126 (27.33%)	24 (22.22%)	102 (28.90%)
	4-6 people	291 (63.12%)	76 (70.37%)	215 (60.91%)
	7 or more people	44 (9.54%)	8 (7.41%)	36 (10.20%)
BMI percentile	Mean (SD)	70.6 (29.2)	64.0 (30.8)	72.7 (28.4)^a^
	Min / Max	0.2 / 99.8	0.2 / 99.7	1.3 / 99.8

^a^indicates significant difference (i.e., two-sided *p* value < 0.05) from the comparison group.

### Associations of KDM physiological age and homeostatic dysregulation with chronological age, race, ethnicity, and household income

**Fig. 1:**
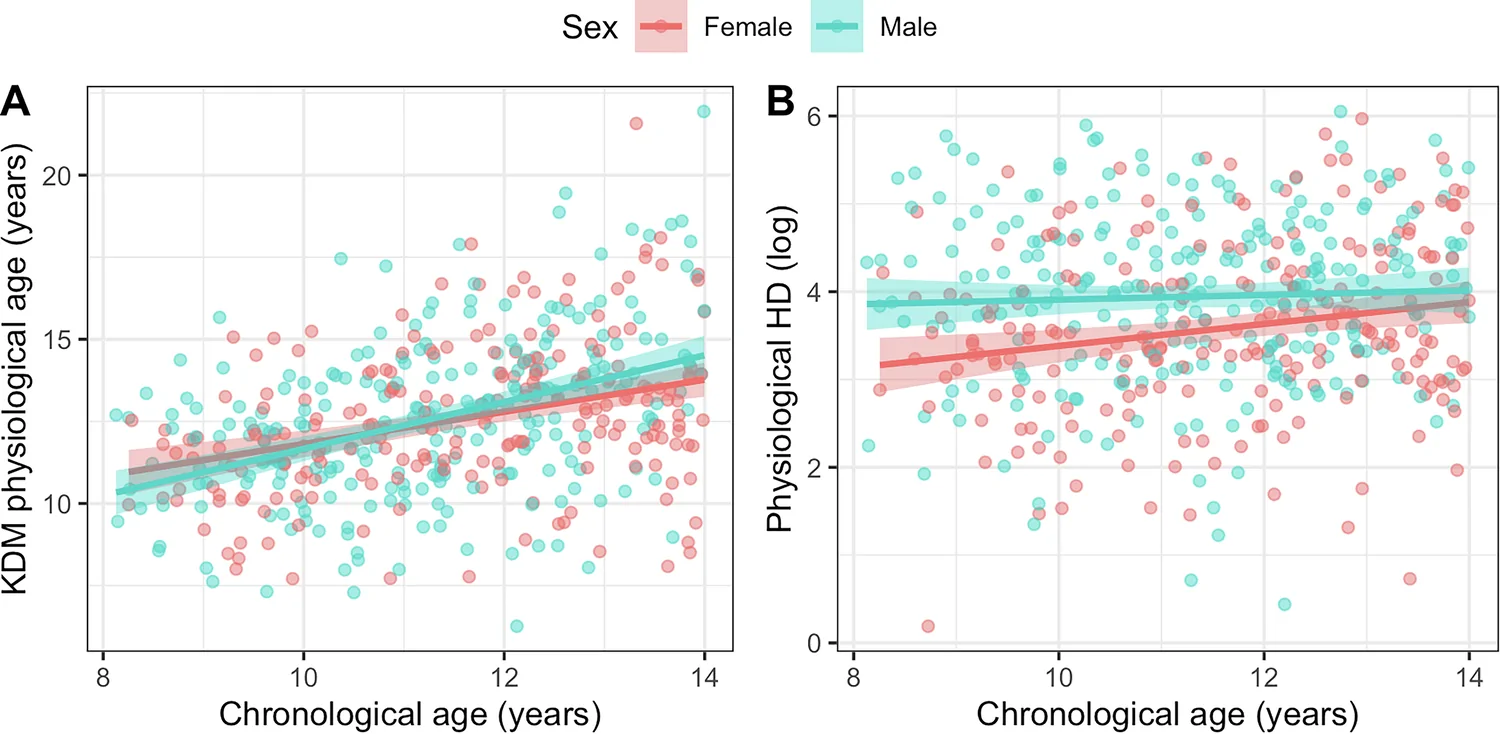
Relationships of KDM physiological age and physiological HD with chronological age in the CHS cohort. **A** KDM physiological age. **B** Physiological HD. KDM Klemera-Doubal Method. HD homeostatic dysregulation.

**Fig. 2:**
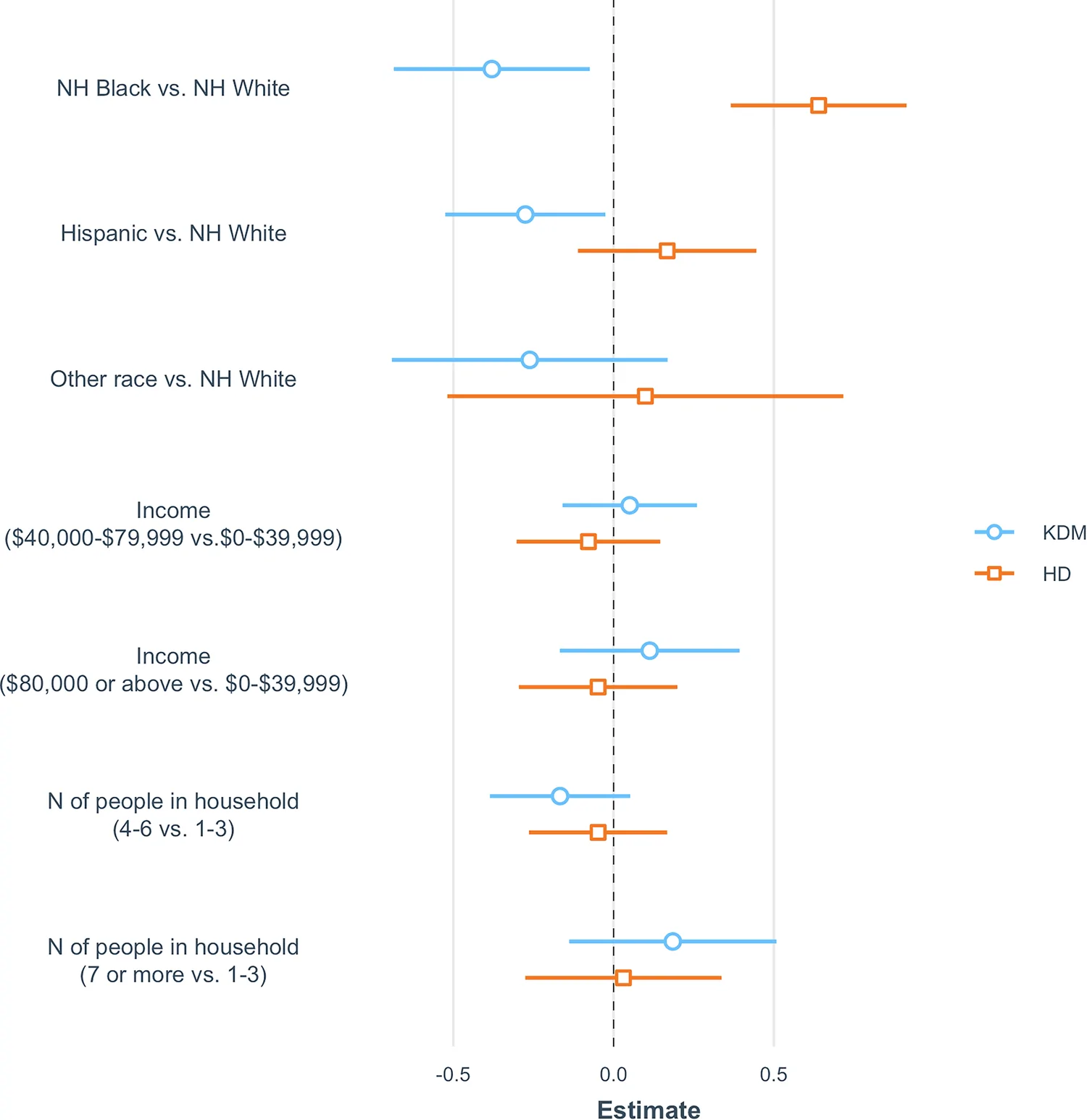
Associations of KDM physiological age and physiological HD with race/ethnicity, household income, and number of people in the household in the CHS cohort. NH non-Hispanic.

KDM physiological age was positively correlated with chronological age (r = 0.38, P < 0.001), and physiological HD had a weak correlation with chronological age (r = 0.09, *P* = 0.043) (Fig. 1), indicating chronological age only explained a low proportion of their variances for each outcome. After controlling for chronological age, neither KDM physiological age residual nor physiological HD residual was correlated with chronological age (r = 0) (Supplementary Fig. 8). Compared to non-Hispanic White children, non-Hispanic Black children (b = –0.38, 95%CI –0.69 to –0.07, *P* = 0.015) and Hispanic children (b = –0.28, 95%CI –0.52 to –0.03, *P* = 0.031) exhibited a younger KDM physiological age. In addition, non-Hispanic Black children showed higher physiological HD compared with non-Hispanic White children (b = 0.64, 95%CI 0.37 to 0.91, P < 0.001). Household income was not associated with KDM physiological age or HD deviations (Fig. 2). The associations of KDM physiological age and HD with non-Hispanic Black race were replicated in NHANES IV validation sample (Supplementary Fig. 9).

### Impact of maltreatment on KDM physiological age

**Fig. 3:**
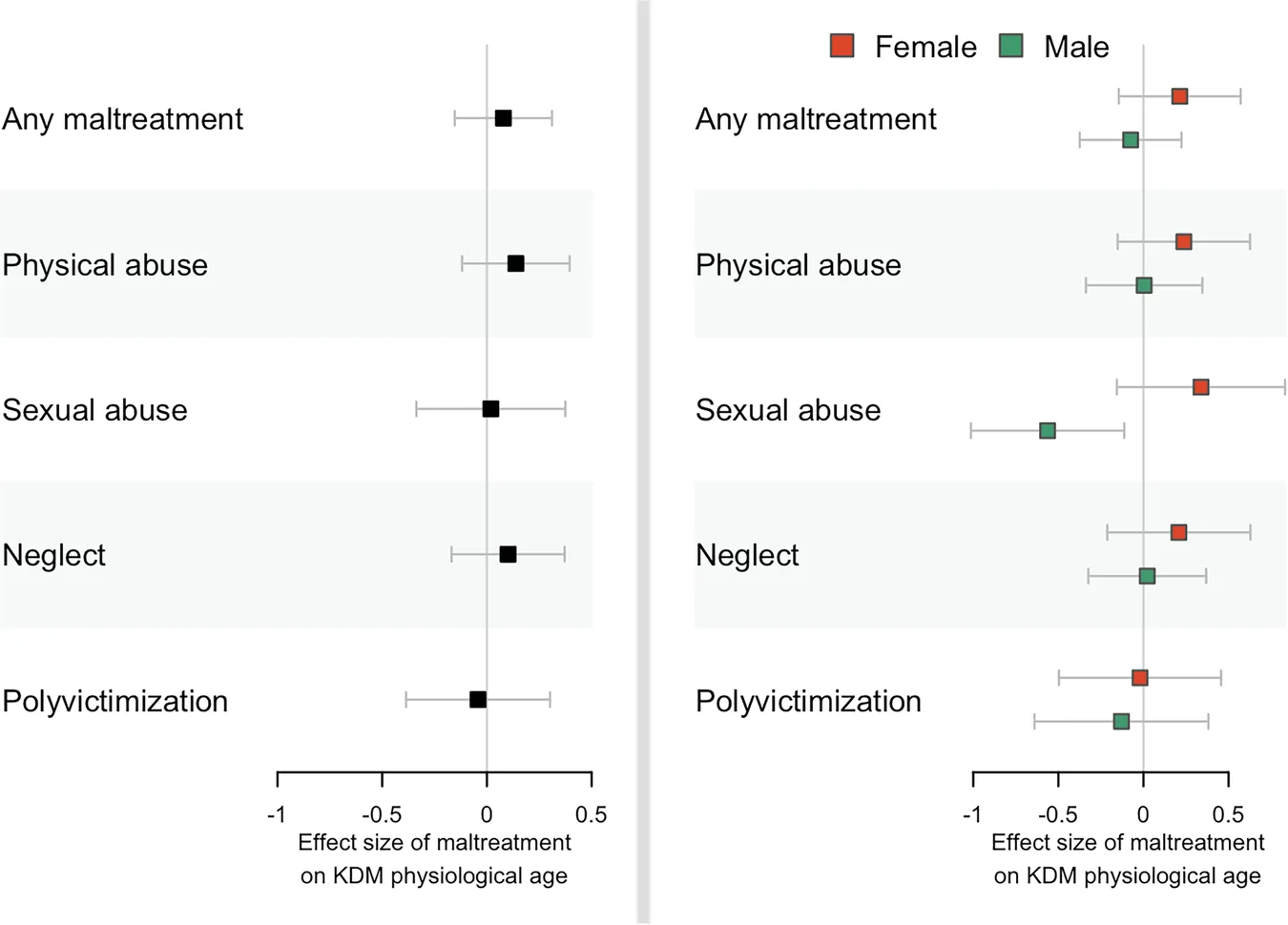
Impact of maltreatment on KDM physiological age in the CHS cohort. The left panel shows the associations in both males and females. The right panel shows the associations by sex. Analyses controlled for sex, race, ethnicity, household income, number of people supported by the household income, and BMI percentile.

No differences in KDM physiological age were observed between children exposed to any form of maltreatment and those unexposed. Maltreatment exposure was further categorized into physical abuse, sexual abuse, neglect, and polyvictimization (i.e., multiple types of maltreatment). Analyses by maltreatment type did not show significant difference in KDM physiological age. The interaction between maltreatment and sex indicated evidence of sex-specific heterogeneity of the impact of sexual abuse (sexual abuse x male: b = –0.92, 95%CI –1.58 to –0.27, *P* = 0.006, adjusted *P* = 0.053). Analyses by sex showed sexual abuse was associated with younger KDM physiological age in males (b = –0.56, 95%CI –1.01 to –0.11, *P* = 0.014) but not in females (b = 0.34, 95%CI –0.16 to 0.83, *P* = 0.180) **(**Fig. 3**)**.

### Impact of maltreatment on physiological homeostatic dysregulation

**Fig. 4:**
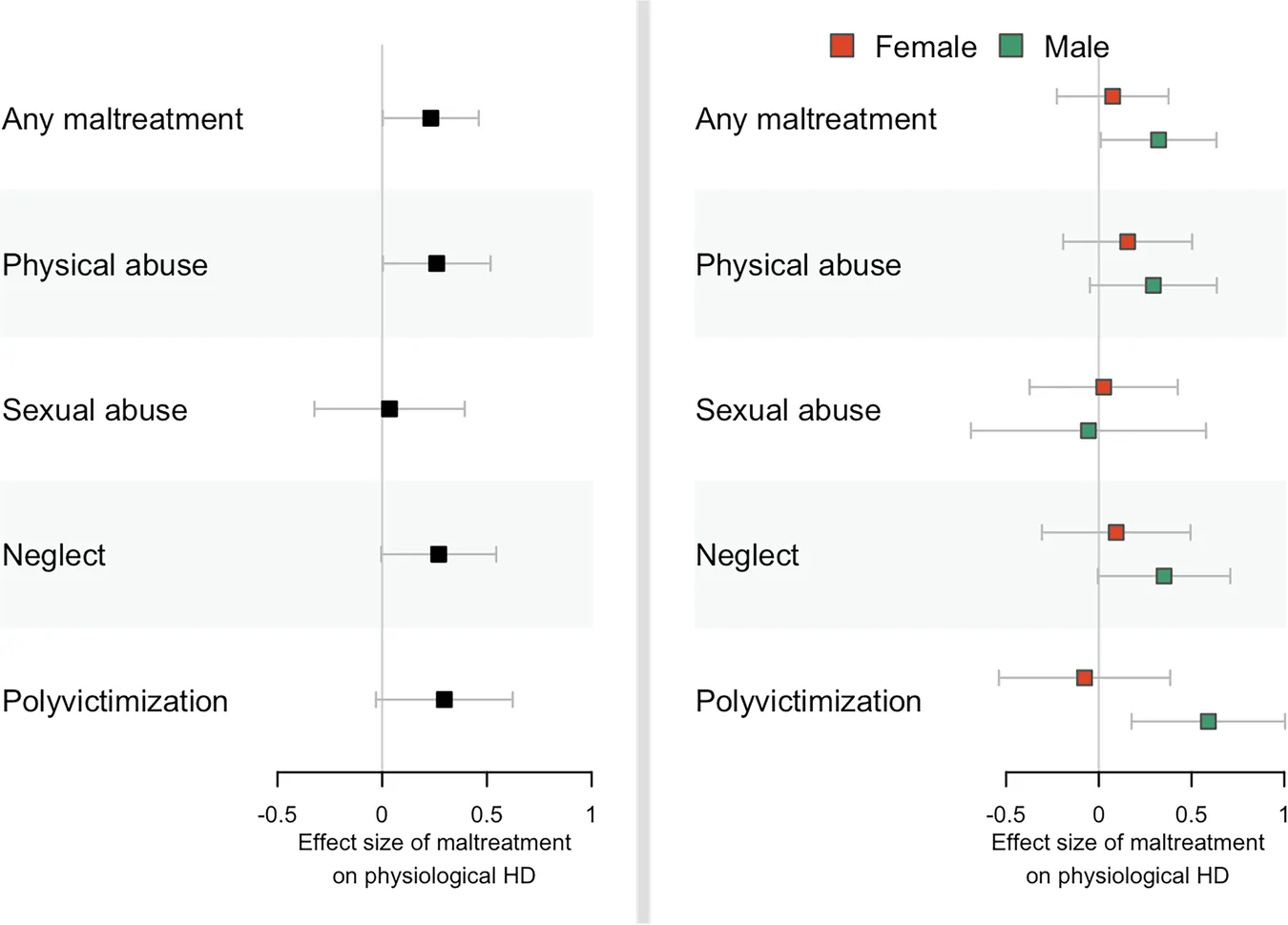
Impact of maltreatment on physiological HD in the CHS cohort. The left panel shows the associations in both males and females. The right panel shows the associations by sex. Analyses controlled for sex, race, ethnicity, household income, number of people supported by the household income, and BMI percentile.

Exposure to any type of maltreatment was associated with greater HD (b = 0.23, 95%CI 0.003 to 0.46, *P* = 0.047, adjusted *P* = 0.122), indicating a greater absolute deviation from a typical developmental profile of 8-13 years. Analyses by specific maltreatment type showed children with physical abuse exhibited greater HD than children without any maltreatment (b = 0.26, 95%CI 0.004 to 0.52, *P* = 0.047, adjusted *P* = 0.122). The interaction between maltreatment and sex indicated evidence of sex-specific heterogeneity of the impact of polyvictimization (polyvictimization x male: b = 0.64, 95%CI 0.07 to 1.21, *P* = 0.028, adjusted *P* = 0.122). Sex-stratified analyses indicated male children exposed to polyvictimization had greater HD than male counterparts without maltreatment (b = 0.59, 95%CI 0.18 to 1.00, *P* = 0.005), whereas the association was not significant among females (b = –0.08, 95%CI –0.54 to 0.38, *P* = 0.744) **(**Fig. 4**)**.

### Sensitivity and exploratory analyses

Sensitivity analyses were conducted by re-calculating KDM physiological age and physiological HD using all possible combinations of included biomarkers, and re-estimating their associations with maltreatment exposure. The point estimates of the associations stabilized as the number of biomarkers increased (Supplementary Figures 10–14). While there were some extreme deviations of effect size estimation for 4- or less biomarker combinations, the effect size estimation was relatively consistent when 5 or more biomarkers were included in KDM or HD calculation.

Additional sensitivity analyses were conducted by controlling for Tanner stage as a covariate when examining the association between maltreatment and KDM physiological age. The effect sizes remained similar, indicating physiological age was a construct independent of pubertal development. This was further supported by the fact that KDM physiological age was weakly correlated with Tanner stage after controlling for chronological age (r = 0.09, *P* = 0.06). In addition, neither binary maltreatment status nor specific maltreatment type was significantly associated with Tanner stage while controlling for chronological age. Analyses by sex indicated physical abuse was associated with advanced Tanner stage in females (b = 0.24, *P* = 0.0496) but not in males.

Exploratory analyses on the impact of frequency and years since the first report of maltreatment on KDM and HD were conducted using the MCS maltreatment variables. Among the 461 participants included in the main analyses, 447 participants had MCS data available. No difference was observed between males and females in terms of maltreatment frequency and years since the first report. With detailed information based on MCS coding, subtle impact of maltreatment on KDM physiological age and physiological HD was revealed. Participants with only 1 report of educational maltreatment had older KDM physiological age but a lower physiological HD (Supplementary Fig. 15). These exploratory results should be interpreted with caution considering that only a small portion of the sample had confirmed or extended histories of specific maltreatment types.

## Discussion

Using data from a prospective cohort study, we assessed the impact of maltreatment exposure on pediatric versions of KDM physiological age and physiological HD among children aged 8-13 years. Exposure to any type of maltreatment was not associated with changes in KDM physiological age but was associated with increased physiological HD. Sexual abuse was associated with younger physiological age in males, indicating delayed physiological development. Physical abuse was linked to elevated HD in combined analyses with both sexes. In addition, exposure to multiple types of maltreatment was associated with elevated HD in males but not in females. However, the findings above would not be considered as statistically significant under FDR < 0.05, and thus should be interpreted with caution.

Although aging has been viewed as the gradual accumulation of stochastic variation and damage in biological systems leading to functional decline [31, 32], certain changes in aging are due to programmed processes that are closely linked with developmental pathways [32–34]. In addition, the lack of a defined boundary between development and aging underscores their continuity and highlights that both represent interconnected facets of a single biological process [12]. Rapid biological development in childhood has been captured by a high rate of biological aging such as rapid shortening of telomere length [35, 36], processes that are exaggerated in conditions such as progeria syndrome [37]. Therefore, the younger physiological age observed among male children with sexual abuse can be interpreted as delayed physiological development or decelerated biological aging compared with non-maltreated youth.

A prior meta-analysis [11] across 54 studies indicated that exposure to early life adversity—including exposure to violence, neglect, and low socioeconomic status—was associated with accelerated pubertal timing and biological markers of cellular aging, such as shortened telomere length and advanced epigenetic age during childhood and adolescence. The meta-analysis also reported that these associations varied by the type of adversity experienced. Although the direction of associations in our study diverged from previous findings, our results similarly indicated that novel associations with physiological age and HD also varied by exposure type. These results underscore the importance of differentiating among maltreatment types when evaluating health outcomes. Directional inconsistencies between our findings and previous studies may reflect fundamental differences between physiological and cellular aging, which represent distinct facets of biological development and aging [21], and may respond differently to adversity. Additionally, a strength of our study was the restriction of the most recent maltreatment exposure to within one year prior to study enrollment, allowing for the assessment of short-term physiological effects. In contrast, most prior studies [19, 20] lacked the ability to capture maltreatment within such a narrow timeframe, which may have contributed to differences in observed associations. Moreover, our exploratory analyses involving maltreatment history beyond the past 12 months indicated that certain types of maltreatment such as educational maltreatment did exhibit effects on accelerated physiological development and aging, although significance depended on the frequency of maltreatment.

The disposable soma theory [38], derived from evolutionary biology, claims that in face of stress and limited resources, energy allocation should prioritize growth and reproduction at the expense of somatic maintenance. Based on this framework, child maltreatment would be expected to accelerate development and reproductive maturation [39]. However, our main findings do not support accelerated physiological development as a short-term consequence of maltreatment. Delayed physiological development as indicated by a younger physiological age was observed among boys exposed to sexual abuse, aligning with prior studies reporting delayed pubertal maturation in maltreated boys [40, 41]. Nonetheless, previous research has also documented accelerated development in girls and null effects in boys, highlighting sex-specific variability [42]. The sex differences could be attributed to differed timing and tempo of physiological development between boys and girls [43], leading to varied vulnerability depending on the overlap between the timing of maltreatment exposure and the timing of physiological development. Sensitivity analyses incorporating Tanner stage indicated that physiological development may involve components distinct from pubertal development. Given that children in our sample were aged 8-13 years (around the typical window for pubertal onset), and that maltreatment was a recent exposure, it is possible these children were already physiologically primed for reproductive maturation, and under stress, somatic development may have been deprioritized. On the other hand, the association between sexual abuse and younger physiological age could imply reverse causation wherein less physiologically matured boys could be more vulnerable to sexual abuse, while physiological maturation may play a less significant role in the likelihood of sexual abuse among girls.

Although physiological HD is traditionally conceptualized as a marker of cumulative wear and tear in the context of aging in adults [16, 22, 44], its application in children should be interpreted as reflecting deviations from typical physiological developmental trajectories. Together, findings with KDM physiological age and HD provide complementary insights into the short-term impact of maltreatment. KDM-based analyses indicated delayed physiological development among males exposed to sexual abuse, and HD-based analyses showed greater deviations from normative physiological development among maltreated children more broadly. These findings underscore the heterogeneity of health outcomes associated with maltreatment and highlight the importance of considering maltreatment type in developmental risk assessments.

We acknowledge limitations. First, the availability of biomarkers in NHANES for children aged 8 to 14 years was limited. Key biomarkers traditionally included in KDM and HD calculations for adults—such as albumin and creatinine, which serve as viable clinical parameters of hepatic and renal function in adolescents—were not consistently available for the full age range in NHANES. Second, only baseline (Visit 1) data from the CHS were analyzed here. Although restricting the most recent maltreatment exposure to within 12 months of enrollment allowed us to focus on short-term physiological responses, we were unable to assess long-term consequences. Follow-up visits from this cohort will enable the examination of within-person longitudinal trajectory models, persistence of physiological dysregulation, and later health outcomes. Third, the majority of the CHS participants were of White and non-Hispanic race or ethnicity, which could limit the generalizability of the findings to other populations. Fourth, children in the CHS maltreatment group were identified based on county Child Welfare Information Systems data. Because participants did not self-identify as having experienced maltreatment and maltreatment history was not explicitly addressed during recruitment, study involvement was not associated with potential stigma related to maltreatment. Children with a history of suspected maltreatment (i.e., who were investigated) per county welfare data, were compared to children without a history of child welfare involvement. This might have attenuated the effect sizes observed in our analyses. However, prior research estimated that 15.4%-65.1% of children without initial record of maltreatment were eventually confirmed with exposure to maltreatment [45]. Including all the suspected cases in the maltreatment group maintained our sample size while minimizing contamination in the comparison group. Our exploratory analyses used MCS variables to examine the impact of two dimensions of maltreatment history including number of previous reports and years since the first report of seven types of maltreatment. Although these exploratory analyses were intended to reveal fine-grained relationships between maltreatment and physiological age and HD, these findings should be interpreted with caution considering the smaller sample size, the lagged nature of reporting, and potential contamination within the comparison group. As the date of maltreatment was unavailable, the date of reporting was used as a rough approximation. Because of the limited sample size, sex differences were not examined with MCS variables. Future research should examine the role of distinct child maltreatment dimensions in greater detail, including the role of more detailed assessments of polyvictimization. Additionally, while our relying on county welfare data in identifying and evaluating maltreatment events could strengthen objectivity, it could also underestimate maltreatment incidence or overlook potential contamination in the comparison group, which might attenuate effect sizes of maltreatment.

In conclusion, among children aged 8 to 13 years, the short-term effects of maltreatment exposure on physiological development and HD varied by maltreatment type and sex. Sexual abuse was associated with delayed physiological development specifically in males, whereas physical abuse was linked to increased HD indicated by combined analyses with both sexes. Exposure to multiple types of maltreatment was also associated with elevated HD among males. These findings highlight the need for early interventions tailored to both the type of maltreatment and the child’s biological sex. Ongoing biannual follow-up of this cohort will provide critical insight into how the physiological effect of maltreatment unfolds over time. As the impact of maltreatment on physiological age and HD was only nominally significant, validation work in other samples is needed.

## Supplementary information

Supplementary Information

## Data Availability

Data of the Child Health Study (CHS) cohort will be available upon reasonable request to and approval by the CHS investigator team. Details about the data request submission procedure can be obtained by contacting the corresponding author – Dr. Idan Shalev (ius14@psu.edu).

## Code availability

R code that used for statistical analyses is available through the Github link without restrictions: https://github.com/zzyyqf/PhysioCompProject.
