# Supplementary material for: Physiological Age and Homeostatic Dysregulation Following Child Maltreatment in Youth
Source: Mol Psychiatry. 2026 May 8;31(10):5586–94. doi: 10.1038/s41380-026-03642-z (PMC13160224; doi:10.1038/s41380-026-03642-z)
Supplement: Supplementary file 1 [file 41380_2026_3642_MOESM1_ESM.docx]

**Supplementary Information for**

**Physiological Age and Homeostatic Dysregulation Following Child Maltreatment in Youth**

Qiaofeng Ye, PhD^1^; Abner T. Apsley, PhD^2^; Laura Etzel, PhD^1^; Waylon J. Hastings, PhD^3,4^; Christopher R. Chiaro, PhD^1^; Hannah M.C. Schreier, PhD^1^; Eric D. Claus, PhD^1^; Alan A. Cohen, PhD^5^; Zachary Fisher, PhD^6^; Christine M. Heim, PhD^7,8,9^; Jennie G. Noll, PhD^10,11^; Chad E. Shenk, PhD, ABPP^10,11^; Idan Shalev, PhD^1^*

*Idan Shalev

Email: ius14@psu.edu

**Supplementary Figure 1. Demographic characteristics of the CHS and the NHANES III cohorts before and after matching.**

**Supplementary Figure 2. Distribution of 27 biomarkers in the CHS and the NHANES III cohorts.**

%BASO, DBP, %MONO, and MPV were excluded because of inconsistent distributions in the two cohorts.

%BASO = Percentage of basophils. %EOSI = Percentage of eosinophils. %LYMP = Percentage of lymphocytes. %MONO = Percentage of monocytes. %NEUT = Percentage of neutrophils. Arm Circum = Arm circumference. BMI = Body mass index. CHOL = Total cholesterol. CHOL/HDLc = Total cholesterol to high density lipoprotein cholesterol ratio. DBP = Diastolic blood pressure. Fe = Iron. HbA1c = Glycated hemoglobin. HCT = Hematocrit. HDLc = High density lipoprotein cholesterol. HGB = Hemoglobin. HR = Heart rate. MCH = mean corpuscular hemoglobin. MCHC = Mean corpuscular hemoglobin concentration. MCV = Mean corpuscular volume. MPV = mean platelet volume. Non-HDLc = Non-high density lipoprotein cholesterol. PLT = Platelet count. RBC = Red blood cell count. RDW = Red cell distribution width. SBP = Systolic blood pressure. WBC = White blood cell. TG = triglyceride.

**Supplementary Figure 3. Correlations between biomarkers and chronological age in the NHANES III cohort.**

**Supplementary Figure 4. Correlation matrix of 14 biomarkers in the NHANES III cohort.**

In constructing this matrix, we excluded biomarkers with inconsistent distributions in CHS and NAHENS III, or with correlations with chronological age less than 0.1.

**Supplementary Figure 5. Correlation matrix in the NHANES III cohort of 9 biomarkers included for physiological age and homeostatic dysregulation calculation.**

**Supplementary Figure 6. Distribution of biomarker values before and after winsorization in the NHANES III cohort. Biomarker values were scaled to the range of 0 to 1 for the convenience of direct comparison.**

Percentages in the brackets are percentages of biomarker values that were winsorized.

**Supplementary Figure 7. Distribution of biomarker values before and after winsorization in the CHS cohort. Biomarker values were scaled to the range of 0 to 1 for the convenience of direct comparison.**

Percentages in the brackets are percentages of biomarker values that were winsorized.

**Supplementary Figure 8. Relationships of KDM physiological age residual and physiological HD residual with chronological age in the CHS cohort.**

**Supplementary Figure 9. Associations of KDM physiological age and physiological HD with race/ethnicity, and income-to-poverty ratio in among participants aged 8-14 years in the NHANES IV cohort (N=8304).**

Similar to NHANES III, NHANES IV was conducted by U.S. Center for Disease Control and Prevention starting Year 1999. Data collected from Year 1999 to 2017 were used in the analyses. Using the same data analytical approach as for NHANES III and CHS, biomarker outliers were winsorized. NHANES IV participants were matched with CHS participants by age, sex, race, and ethnicity. Income-to-poverty ratio is the ratio of family income to poverty based on the “Department of Health and Human services” poverty guidelines. Higher income-to-poverty ratio indicates higher relative family income in that year. Values of income-to-poverty ratio at or above 5.00 were coded as 5.00.

**Supplementary Figure 10. Sensitivity analyses showing the change of effect size estimates of any type of maltreatment with number of biomarkers included in KDM physiological age and physiological HD calculation in the CHS cohort.**

**Supplementary Figure 11. Sensitivity analyses showing the change of effect size estimates of physical abuse with number of biomarkers included in KDM physiological age and physiological HD calculation in the CHS cohort.**

**Supplementary Figure 12. Sensitivity analyses showing the change of effect size estimates of sexual abuse with number of biomarkers included in KDM physiological age and physiological HD calculation in the CHS Cohort.**

**Supplementary Figure 13. Sensitivity analyses showing the change of effect size estimates of neglect with number of biomarkers included in KDM physiological age and physiological HD calculation in the CHS cohort.**

**Supplementary Figure 14. Sensitivity analyses showing the change of effect size estimates of polyvictimization with number of biomarkers included in KDM physiological age and physiological HD calculation in the CHS cohort.**

**Supplementary Figure 15. Impact of maltreatment frequency and years since the first report based on MCS coding on KDM physiological age and physiological HD in the CHS cohort.**

The effect sizes of the seven types of maltreatment were adjusted for each other in the models. Covariates included sex, race, ethnicity, household income, number of people supported by the household income, and BMI percentile.

**Supplementary Table 1. Demographic characteristics of the NHANES III cohort before matching (N = 689).**

| **Variable** | **Statistical Type/Levels** | **Statistics** |
| --- | --- | --- |
| Age | Min / Max | 8.0 /14.0 |
|  | Mean (SD) | 11.0 (1.7) |
| Sex | Female | 339 (49.20%) |
|  | Male | 350 (50.80%) |
| Race | White | 352 (51.09%) |
|  | Black | 308 (44.70%) |
|  | Other | 29 (4.21%) |
| Ethnicity | Non-Hispanic | 446 (64.73%) |
|  | Hispanic | 243 (35.27%) |

**Supplementary Table 2. Demographic characteristics of the CHS participants included and excluded from analyses.**

| **Variable** | **Statistic Type/Levels** | **Excluded (N=237)** | **Included (N=461)** | **P value** |
| --- | --- | --- | --- | --- |
| Age | Mean (SD) | 10.9 (1.6) | 11.4 (1.5) | <0.0001 |
|  | Min / Max | 8.1 / 14.0 | 8.1 / 14.0 |  |
| Sex | Female | 133 (56.12%) | 216 (46.85%) | 0.0205 |
|  | Male | 104 (43.88%) | 245 (53.15%) |  |
| Race | White | 138 (58.47%) | 353 (76.57%) | <0.0001 |
|  | Black or African American | 41 (17.37%) | 61 (13.23%) |  |
|  | Other | 57 (24.15%) | 47 (10.20%) |  |
|  | Missing | 1 | 0 |  |
| Ethnicity | Non-Hispanic | 196 (84.85%) | 401 (86.98%) | 0.4413 |
|  | Hispanic | 35 (15.15%) | 60 (13.02%) |  |
|  | Missing | 6 | 0 |  |
| Household income | $0-$39,999 | 109 (48.02%) | 234 (50.76%) | 0.7730 |
|  | $40,000-$79,999 | 66 (29.07%) | 130 (28.20%) |  |
|  | $80,000 or above | 52 (22.91%) | 97 (21.04%) |  |
|  | Missing | 10 | 0 |  |
| Number of people  in the household | 1-3 people | 67 (30.59%) | 126 (27.33%) | 0.6267 |
|  | 4-6 people | 130 (59.36%) | 291 (63.12%) |  |
|  | 7 or more people | 22 (10.05%) | 44 (9.54%) |  |
|  | Missing | 18 | 0 |  |
| Maltreatment type | Comparison | 66 (27.85%) | 108 (23.43%) | 0.0685 |
|  | Physical abuse | 45 (18.99%) | 123 (26.68%) |  |
|  | Sexual abuse | 36 (15.19%) | 53 (11.50%) |  |
|  | Neglect | 68 (28.69%) | 119 (25.81%) |  |
|  | Polyvictimization | 22 (9.28%) | 58 (12.58%) |  |
| BMI percentile | Mean (SD) | 70.3 (31.4) | 70.6 (29.2) | 0.5544 |
|  | Min / Max | 0 / 99.7 | 0.2 / 99.8 |  |
|  | Missing | 4 | 0 |  |
